# Supplementary material for: Agarose-Degrading Characteristics of a Deep-Sea Bacterium Vibrio Natriegens WPAGA4 and Its Cold-Adapted GH50 Agarase Aga3420
Source: Mar Drugs. 2022 Nov 1;20(11):692. doi: 10.3390/md20110692 (PMC9698624; doi:10.3390/md20110692)
Supplement: Supplementary file 1 [file marinedrugs-20-00692-s001.zip › marinedrugs-1886492-supplementary.pdf]

Table S1. Comparison of the properties of rAga3420 and other GH50 agarases

Family	Bacterium	Isolation sites	Protein name	Optimal temperature (°C)	Optimal pH	Cold-adapted property	Reference
GH50	*V.natriegens* WPAGA4	Deep-sea sediment	Aga3420	40	7.0	0°C; >40%	This study
GH50	*Agarivorans albus* YKW-34	Turban shell	AgaA34	40	8.0	10°C; >20%	1
GH50	*A. gilvus* WH0801	Seaweed	AgWH50A	30	6.0	20°C; >20%	2
GH50	*A. gilvus* WH0801	Seaweed	AgWH50C	30	6.0	20°C; >10%	3
GH50	*A.* sp. JAMB-A11	Sediment	AgaA11	40	7.5-8.0	20°C; 20%	4
GH50	*Paenibacillus agarexedens* BCRC 16000	Soil	AgaB-4	55	6.0	20°C; >40%	5
GH50	*Pseudoalteromonas* sp. NJ21	Sediment	Aga21	30	8.0	10°C; >85%	6
GH50	*Saccharophagus degradans* 2-40	Marine bacterium	Aga50D	30	7.0	20°C; >80%	7
GH50	*Streptomyces coelicolor* A3(2)	Soil	DagB	40	7.0	20°C; 20%	8
GH50	*Victivallis vadensis* ATCC BAA-548	Human fecal	VadG925	40, 60	7.0	4°C; >20%	9

N.D. not determined

Table S2. The values of ANI and DDH of *Vibrio natriegens* WPAGA4 with other Vibrio species.

Bacterium	ANI	dDDH
V. natriegens NBRC 15636	98.23	84.6
V. chemaguriensis Iso1	80.74	23.4
V. hyugaensis 090810a	80.56	22.3
V. diabolicus CNCM I-1629	79.65	23.4
V. inhibens CECT 7692	79.51	22.6
V. communis LMG 25430	79.00	22.7
V. jasicida CAIM 1864	76.97	22.6
V. campbellii CAIM 519	76.67	22.8
V. nereis NBRC 15637	74.44	22.4

Table S3. The dbCAN annotation result of the 4 putative agarase genes

Gene ID	EC#	GH family	Amino acid location of the domain
aga3418	3.2.1.81	GH50	281-963
aga3419	3.2.1.81	GH50	251-919
aga3420	3.2.1.81	GH50	270-948
aga3421	3.2.1.81	GH50	69-739

Table S4. The primer pairs for the putative agarase gene amplification

Gene ID	Forward primer (5'-3')	Reverse primer (5'-3')
aga3418	GCTACAAAAACACCAGACACATCC	TTTGTTAATAGATCCGAATCGTCG
aga3419	TCAACGTTAGTGACTTCTTTTGAAAG	TGATACGTATTTTCTACGTACCCATC
aga3420	TGTCAATCGACGACAGGATC	TTTAAAGCGGTTGTTATAGAGGTTTG
aga3421	ACACCAACTATAAATGATGTGGTTAG	CTTATAACGCCGTTTATACATACAGC

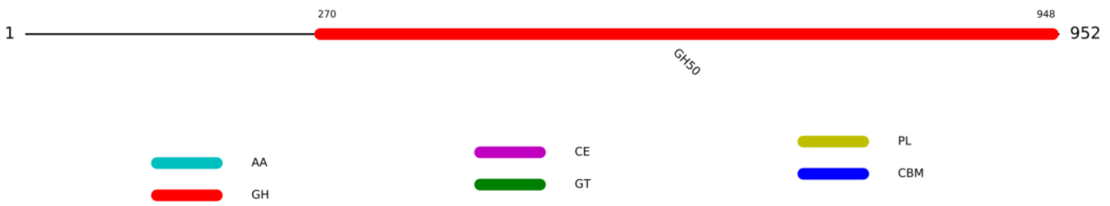

Fig. S1. The dbCAN annotation result of aga3420

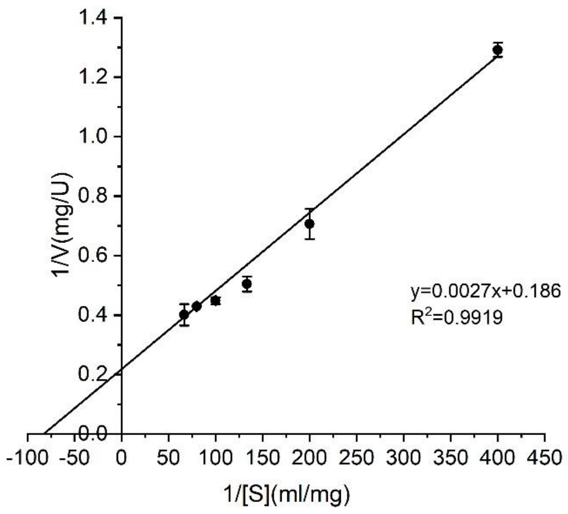

Fig. S2. Lineweaver-Burk plot of the kinetic parameter determination of rAga3420

References

1. Fu, X.; Lin, H.; Kim, S.M. Purification and characterization of a novel beta-agarase, AgaA34, from Agarivorans albus YKW-34. *Appl Microbiol Biotechnol.* **2008**, 78, 2, 265-73.

2. Liu, N.; Mao, X.; Du, Z.; Mu, B.; Wei, D. Cloning and characterisation of a novel neoagarotetraose-forming-β-agarase, AgWH50A from Agarivorans gilvus WH0801. *Carbohydr Res*. **2014**, 388, 147-51.
3. Liu, N.; Mao, X.; Yang, M.; Mu, B.; Wei, D. Gene cloning, expression and characterisation of a new β-agarase, AgWH50C, producing neoagarobiose from Agarivorans gilvus WH0801. *World J Microbiol Biotechnol.* **2014**, 30, 6, 1691-8.
4. Ohta, Y.; Hatada, Y.; Ito, S.; Horikoshi, K. High-level expression of a neoagarobiose-producing beta-agarase gene from Agarivorans sp. JAMB-A11 in Bacillus subtilis and enzymic properties of the recombinant enzyme. *Biotechnol Appl Biochem.* **2005**, 41,Pt 2:183-91.
5. Chen, Z.W,; Lin, H.J.; Huang, W.C.; Hsuan, S.L.; Lin, J.H.; Wang, J.P. Molecular cloning, expression, and functional characterization of the β-agarase AgaB-4 from Paenibacillus agarexedens. *AMB Express.* **2018,** 8, 1, 49.
6. Li, J.; Hu, Q.; Li, Y.; Xu, Y. Purification and characterization of cold-adapted beta-agarase from an Antarctic psychrophilic strain. *Braz J Microbiol.* **2015**, 46, 3, 683-90.
7. Kim, H.T.; Lee, S.; Lee, D.; Kim, H.S.; Bang, W.G.; Kim, K.H.; Choi, I.G. Overexpression and molecular characterization of Aga50D from Saccharophagus degradans 2-40: an exo-type beta-agarase producing neoagarobiose. *Appl Microbiol Biotechnol.* **2010**, 86, 1, 227-34.
8. Temuujin, U.; Chi, W.J.; Chang, Y.K.; Hong, S.K. Identification and biochemical characterization of Sco3487 from Streptomyces coelicolor A3(2), an exo- and endo-type β-agarase-producing neoagarobiose. *J Bacteriol.* **2012**, 194, 1, 142-9.
9. Temuujin, U.; Chi, W.J.; Park, J.S.; Chang, Y.K.; Song, J.Y.; Hong, S.K. Identification and characterization of a novel β-galactosidase from Victivallis vadensis ATCC BAA-548, an anaerobic fecal bacterium. *J Microbiol.* **2012**, 50, 6, 1034-40.
